# A novel nanobody as therapeutics target for EGFR-positive colorectal cancer therapy: exploring the effects of the nanobody on SW480 cells using proteomics approach

**DOI:** 10.1186/s12953-022-00190-6

**Published:** 2022-05-16

**Authors:** Thomanai Lamtha, Sucheewin Krobthong, Yodying Yingchutrakul, Pawitrabhorn Samutrtai, Christopher Gerner, Lueacha Tabtimmai, Kiattawee Choowongkomon

**Affiliations:** 1 Department of Biochemistry, Faculty of Science, Laboratory of Protein Engineering and Bioinformatics, Kasetsart University, Ngam Wong Wan, Chatuchak, Bangkok, 10900 Thailand; 2 Department of Biochemistry, Faculty of Science, Mahidol University, Bangkok, Thailand; 3 Center for Neuroscience, Faculty of Science, Mahidol University, Bangkok, 10400 Thailand; 4 National Omics Center, NSTDA, Pathum Thani, 12120 Thailand; 5 Department of Pharmaceutical Sciences, Faculty of Pharmacy, Chiang Mai University, Chiang Mai, 50200 Thailand; 6 Department of Analytical Chemistry, University of Vienna, Währinger Straße 38, 1090 Vienna, Austria; 7 Department of Biotechnology, Faculty of Applied Science, King Mongkut’s University of Technology North Bangkok, Bangkok, 10800 Thailand

**Keywords:** Antibodies, Gefitinib, SW480, Tyrosine kinase inhibitors, Protein interactions, LC–MS/MS

## Abstract

**Background:**

The epidermal growth factor receptor (EGFR) overexpression is found in metastatic colorectal cancer (mCRC). Targeted molecular therapies such as monoclonal antibodies (mAbs) and tyrosine kinase inhibitors (TKI) are becoming more precise, targeting specifically for cancer therapeutics. However, there are adverse effects of currently available anti-EGFR drugs, including drug-resistant and side effects. Nanobodies can overcome these limitations. Our previous study has found that cell-penetrable nanobodies targeted at EGFR-tyrosine kinase were significantly reduced EGFR-positive lung cancer cells viability and proliferation. The aim of the present study was to determine the effect of cell-penetrable nanobody (R9VH36) on cell viability and proteomic profile in EGFR-positive human colorectal cancer cell lines.

**Methods:**

The human colorectal carcinoma cell line (SW480) was treated with R9VH36, compared with gefitinib. Cell viability was monitored using the MTT cell viability assay. The proteomic profiling was analyzed by LC–MS/MS .

**Results:**

The half-maximal inhibitory concentration (IC_50_) values determined for R9VH36 and gefitinib against SW480 were 527 ± 0.03 nM and 13.31 ± 0.02 μM, respectively. Moreover, both the gefitinib-treated group and nanobody-treated group had completely different proteome profiles. A total 6626 differentially expressed proteins were identified. PCA analysis revealed different proteome profiling in R9VH36 experiment. There were 8 proteins in R9VH36 that significantly exhibited opposite expression directions when compared to gefitinib. These proteins are involved in DNA-damage checkpoint processes.

**Conclusion:**

The proteomics explored those 6,626 proteins had different expressions between R9VH36 and gefitinib. There were 8 proteins in R9VH36 exhibited opposite expression direction when comparing to gefitinib. Our findings suggest that R9VH36 has the potential to be an alternative remedy for treating EGFR-positive colon cancer.

**Supplementary Information:**

The online version contains supplementary material available at 10.1186/s12953-022-00190-6.

## Background

Metastatic colorectal cancer (mCRC) is the last stage of colorectal which is incurable, the most common site of metastases for colon or rectal cancer is the liver [1]. For a decade, retrospective analysis had developed various strategies for identifying a novel cancer biomarker [2]. Reportedly, 60–80% of mCRC have been associated with Epidermal Growth Factor Receptor (EGFR) alteration. Overexpression and mutation of EGFR reflect cancer progression and prognosis [3, 4]. A novel target therapy remains a challenge. EGFR is a glycoprotein receptor that belongs to the HER family (HER1(EGFR), HER2-neu, HER3, and HER4). EGFR plays a crucial role in cell proliferation, cell differentiation, and cell migration. Structural EGFR consist of 3 major domain which are the extracellular domain (ECD), transmembrane helix (TM), and intrinsic tyrosine kinase domain (TK) [5, 6]. EGFR-targeted therapy has been developed based on EGFR structure, EGFR inhibitors have divided into the monoclonal antibody (mAb) and small-molecule inhibitor (SM) [7, 8]. Accordingly, EGFR has its cognate ligands which and bind to ECD to trigger receptor dimerization leading to trigger intracellular signaling; called EGFR activation. Among treatment options for metastatic colorectal cancer (mCRC), cetuximab and panitumumab are two distinct monoclonal antibodies (mAbs) targeting the EGFR, currently indicated for the same subgroup of patients, those with RAS wild-type (wt) metastatic disease [9, 10]. However, TK mutations have been concerned which led to ligand-independent activation [11, 12]. SM has been developed as ATP-analogue to prevent ATP binding at TK; called TKI. Approved TKI (Gefitinib, Erlotinib, Lapatinib) have been widely used in the clinical phase, and are effective in various cancer such as lung, breast, prostate, including colorectal cancer [13]. Unfortunately, long-term exposed TKI developed drug-resistant occurrence [14]. Cetuximab has been used in colorectal treatment, but 50–75% of patients are responders. The remaining patients were diagnosed as non-responder which have been found KRAS mutation. The benefit of cetuximab was limited to patients with KRAS wild-type tumors [15]. There might be an additional mechanism leading to an escape from the treatment. Although, gefitinib is effective in EGFR-expressing cancer cells but provokes innate resistance via nuclear factor-kappa B (NF-κB) signaling. Therefore, EGFR-targeting therapy remains the current therapeutics target for cancer therapy.

Herein, antibody fragments become a new strategy in target therapy development [16, 17]. According to the smallest form, they are able to resist harsh conditions, pH-stability, and also target the hidden epitope [18]. Some research has developed antibody fragments against EGFR for reducing cancer growth. CONAN-1 which mimics the paratope of Cetuximab and Matuzumab completely inhibits EGF-dependent cell proliferation and reduces tumor size in an in vivo [19]. EGF-competitive nanobody which is derived from camelid animals is effective in both in vitro and in vivo [19]. However, the mechanism of these antibody fragments remains unclear. Therefore, proteomic profiling was performed to monitor protein dynamics after treatment. An EGFR-tyrosine kinase (EGFR-TK)-targeted cell penetrable nanobody or R9VH36 which previously demonstrated cytotoxicity on the cell harboring KRAS mutations was used to compare with gefitinib on the colorectal cell line, SW480 (G12V) [20]. We hypothesized that the others EGFR-positive KRAS mutant cancers were affected similar with EGFR-positive KRAS mutant lung cancers treated with R9VH36 and might reveal the different mechanism between the R9VH36 and EGFR-TKI. The results showed that R9VH36 reduced EGFR-positive mCRC viability and revealed distinct quantitative and qualitative protein characteristics to gefitinib treatment.

## Materials and methods

### Cell culture

SW480 (human colorectal carcinoma overexpressing EGFR) was kindly gifted by Dr. Michael Jakupec (Department of Inorganic Chemistry, University of Vienna, Austria). The cells were cultured as adherent monolayers in plastic tissue culture dishes in complete growth medium Eagle's Minimum Essential Medium (EMEM) (ATCC, USA) supplemented with 10% (v/v) heat-inactivated calf bovine serum (ATCC, USA) and 100 U/mL antibiotics (Sigma-Aldrich, USA), 0.1 mM non-essential amino acid and 1 mM sodium pyruvate. NIH/3T3 (EGFR-negative mouse fibroblast) was kindly gifted by Dr. Chomdao Sinthuvanich (Department of Biochemistry, Faculty of Science, Kasetsart University, Thailand). The cells were cultured in DMEM (Gibco, Carlsbad, CA, USA) containing 10% fetal bovine serum (ATCC, USA) and 100 U/mL antibiotics (Sigma-Aldrich, USA). Cells were maintained at 37ºC in a humidified incubator in an atmosphere with 5% CO_2_ and replenished every 3 days before the experiment set up. The cells were maintained at 37ºC in a humidified incubator in an atmosphere with 5% CO_2_.

### Cell viability assay

The R9VH36-nanobody was dissolved in PBS at the concentration of 100 µM. Gefitinib (Santa Cruz Biotechnology, USA) was dissolved in DMSO (Loba Co., India) at the concentration of 10 mM. The final concentration was obtained by dilution in a complete culture medium. The cancer cell lines viability assay was performed using MTT assay (PanReac AppliChem, Germany) according to the manufacturer’s instructions. SW480 and NIH/3T3 cells were seeded on 96-well microtiter plates (5 × 10^5^ cells/mL) in 100 μL of complete growth medium and allowed to attach overnight at 37 °C, 5% CO_2_. The next day, the cultured cells were treated with varying concentrations of R9VH36 (two-fold dilutions, starting at 10 µM) and gefitinib (two-fold dilutions, starting at 100 µM) for 72 h, 100 μL/well MTT reagent (0.5 mg/mL) was added to each well and incubated for an additional 4 h at 37ºC. The medium containing non-metabolized MTT was then aspirated, and 50 μL DMSO was added to solubilize the reduced formazan product. Finally, the absorption was then measured at excitation and emission wavelengths of 550 and 590 nm, respectively by a microplate reader (BioTek Synergy HTX, U.S.A.). Based on spectrophotometric measurements, the cell viability was calculated compared to the control cells (the absorbance of the control cells as 100% viability). The 50% inhibitory concentration (IC_50_) value was calculated from the graph of the dose–response by the following equation,

$$y=Bottom+\left(\frac{Top-Bottom}{1+{10}^{LogIC50-X}}\right)$$

where Bottom is the minimum values, Top is the maximum values, y is the cell index, and X is the testing concentration in logarithm unit.

Three independent experiments were performed with triplicates-treatment preparation. The IC50 of testing samples was used in the proteomics analysis.

Sample preparation for proteomics analysis.

The cells were placed in a T25 flask (Nest, U.S.A.) (~ 10^7^ cells/flask) in triplicates. The day after, the cells were then replaced with a fresh medium containing 10 nM of R9VH36 or gefitinib for 1 h. The treated cell lysates were lysed in lysis buffer (10 mM HEPES/NaOH, pH 7.4, 0.25 M sucrose, 10 mM NaCl, 3 mM MgCl_2_, 0.5% Triton X-100) supplemented with a protease inhibitor cocktail (Sigma-Aldrich Co., USA). The supernatant was collected by centrifugation at 12000* g*. and subsequent to ice-cold acetone precipitation. After precipitation, all samples were reconstituted in sample buffer (6 M Urea, 2 M Thiourea, 0.05% SDS, 10 mM NaCl). The protein solution was diluted in 10 mM ammonium bicarbonate at 1:20 ratio (v/v). The total protein (25 µg) was subjected to gel-free based digestion. Next, sulfhydryl bond reduction was performed using 5 mM DTT (Sigma Aldrich Co.) in 10 mM ammonium bicarbonate at 25 °C for 3 h and sulfhydryl alkylation using IAA (Sigma Aldrich Co.) at room temperature for 30 min in the dark. All samples were enzymatically digested for 16 h. The tryptic peptides were cleaned-up using C18 Zip-tip (Merck Millipore, USA) and reconstituted in 0.1% formic acid before being subjected to LC–MS/MS.

### LC–MS/MS setting for tryptic peptide analysis

The tryptic peptides were analyzed using tandem mass spectrometers, Orbitrap HF hybrid mass spectrometer combined with an UltiMate 3000 LC system (Thermo Fisher, USA). The tryptic peptides were first desalted on the line of a reverse-phase C18 PepMap 100 trapping column, before being resolved onto a C18 PepMapTM 100 capillary column with a 135-min gradient of CH_3_CN, 0.1% formic acid, at a flow rate of 300 nL/min. Peptides were analyzed by applying a data-dependent top10 method consisting of a scan cycle initiated by a full scan of peptide ions, followed by high-energy collisional dissociation and MS/MS scans on the 10 most abundant precursor ions. Full scan mass spectra were acquired from *m/*z 400 to 1600 with an AGC target set at 3 × 10^6^ ions and a resolution of 70,000. MS/MS scan was initiated when the ACG target reached 10^5^ ions. Ion selection was performed applying a dynamic exclusion window of 15 s.

Raw files were analyzed by the Proteome Discoverer software version. 2.4 (Thermo Scientific) using the SEQUEST, Percolator, and Minora algorithms. LC–MS spectrum was matched against the UniProtKB reviewed database (11/05/2021). For protein identification and quantification, the setting parameters were as follows: a maximum of two trypsin missed cleavages were allowed with a precursor mass tolerance of 20 ppm and fragment mass tolerance of 0.01 Da. Carbamidomethylation + 57.021 Da (Cysteine) was selected as static modifications and oxidation + 15.995 Da (Methionine) was selected as dynamic modifications. The Fase discovery rate (FDR) of peptide and protein identification were both set to 0.05. The normalization of relative protein abundances ratio was performed by total peptide amount for each LC-runs (across all runs; *n* = 18) by normalization algorithm of Proteome discoverer software. To assembly differential expressed protein list, multiple consensus workflows were used within the Proteome Discoverer software to assemble the PSMs into peptide groups, protein database matches, and finally, non-redundant proteins groups using the principle of strict parsimony as defined by the vendor software defaults. The proteomics data have been deposited to the ProteomeXchange Consortium via the PRIDE partner repository with the dataset identifier PXD033149 and 10.6019/PXD033149 [21]. Quality control (QC) assessment including variation in TIC and peptide–spectrum match efficiency was evaluated [22]. The number of PSM was estimated by FDR values. The data passed the QC were subjected to downstream analysis.

### Statistical analysis

Data are presented as the mean ± SEM. Statistical significance was analyzed by one-way ANOVA using the GraphPad Prism software (version 7.0, CA, USA). All experiments were carried out with three independent replicates (*n* = 3). One-way analysis of variance (one-way ANOVA) was performed using PD for proteomics software. The significance in differences was determined by Duncan’s multiple range test (*p* < 0.05).

## Results

### SW480 cell viability

Cell viability was determined by MTT assay using logarithmically growing SW480 cells treated with various concentrations of R9VH36 or gefitinib. Following 72 h of continuous exposure to the R9VH36 or gefitinib, the cell lines showed a dose-dependent inhibition of the cell growth.

**Fig. 1:**
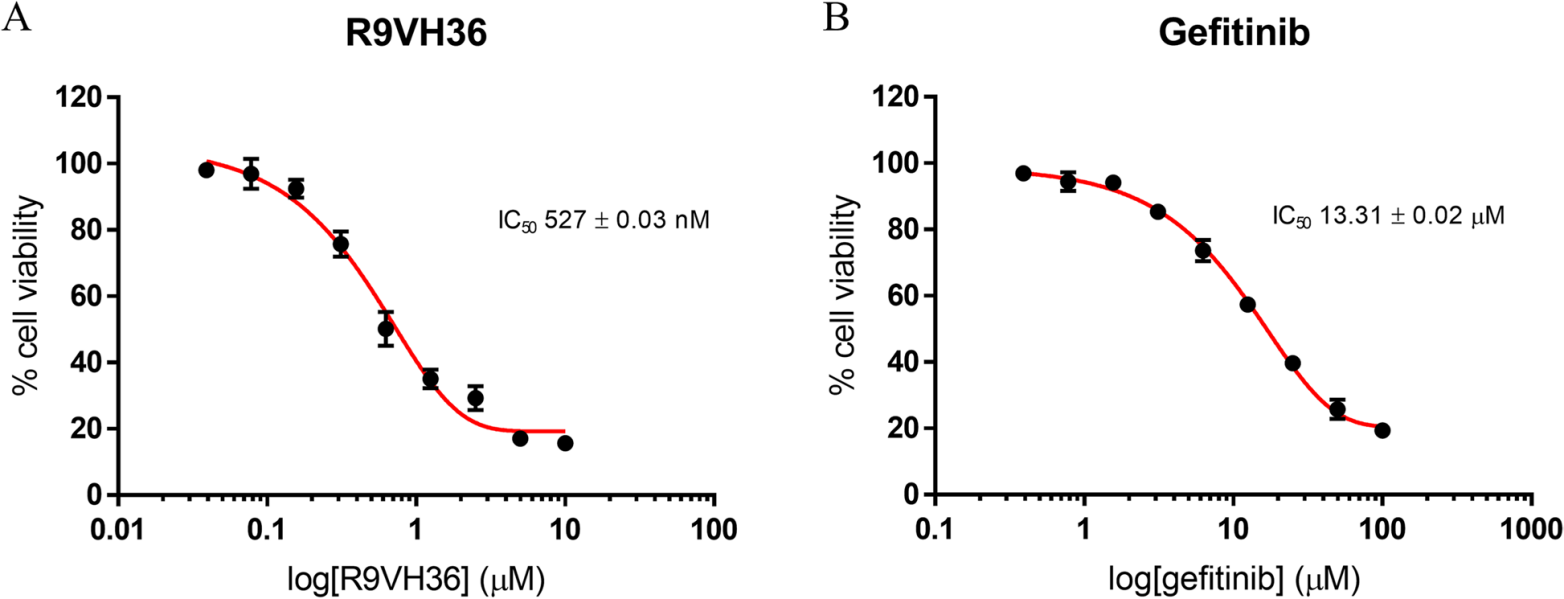
Cell viability of gefitinib and R9VH36 treatments in the SW480 cells. The cells were treated with various concentrations of the nanobodies (**A**) and gefitinib (**B**) for 3 days. The results are expressed as mean ± SD of triplicate experiments

A concentration range of R9VH36 at 0.1–1 μM showed slight toxicity to the cells (cell survival rate > 90%). The estimated VH36 and gefitinib IC_50_ values were 527 ± 0.03 nM and 13.31 ± 0.02 µM for SW480, respectively (Fig. 1A and B). Our results revealed superior cytotoxicity of R9VH36 to A549 cells, having a more than twofold increase in the cytotoxic activity compared with gefitinib which is a well-known drug. These results suggested us the specificity of R9VH36 for the EGER-expressing SW480. Moreover, low cell cytotoxicity was observed in the control NIH/3T3 cells (supplementary Fig. 1). R9VH36 also demonstrates the cytotoxic effect on the EGFR-positive lung cell harboring KRAS mutations (G12S), A549. In this study, we showed that R9VH36 also inhibits cell viability on the other KRAS mutations metastatic colorectal cancer, SW480 (G12V). Additionally, we found that the other EGFR-positive KRAS mutations colorectal cancer, SW620(G12V), and HCT116(G13D), cell viability was affected by R9VH36 treatment (supplementary Fig. 2). These results demonstrated the biological effects of the cell-penetrable R9VH36 treatment on EGFR-positive metastatic colorectal cancer.

### Proteome profiling of SW480 cells

**Fig. 2:**
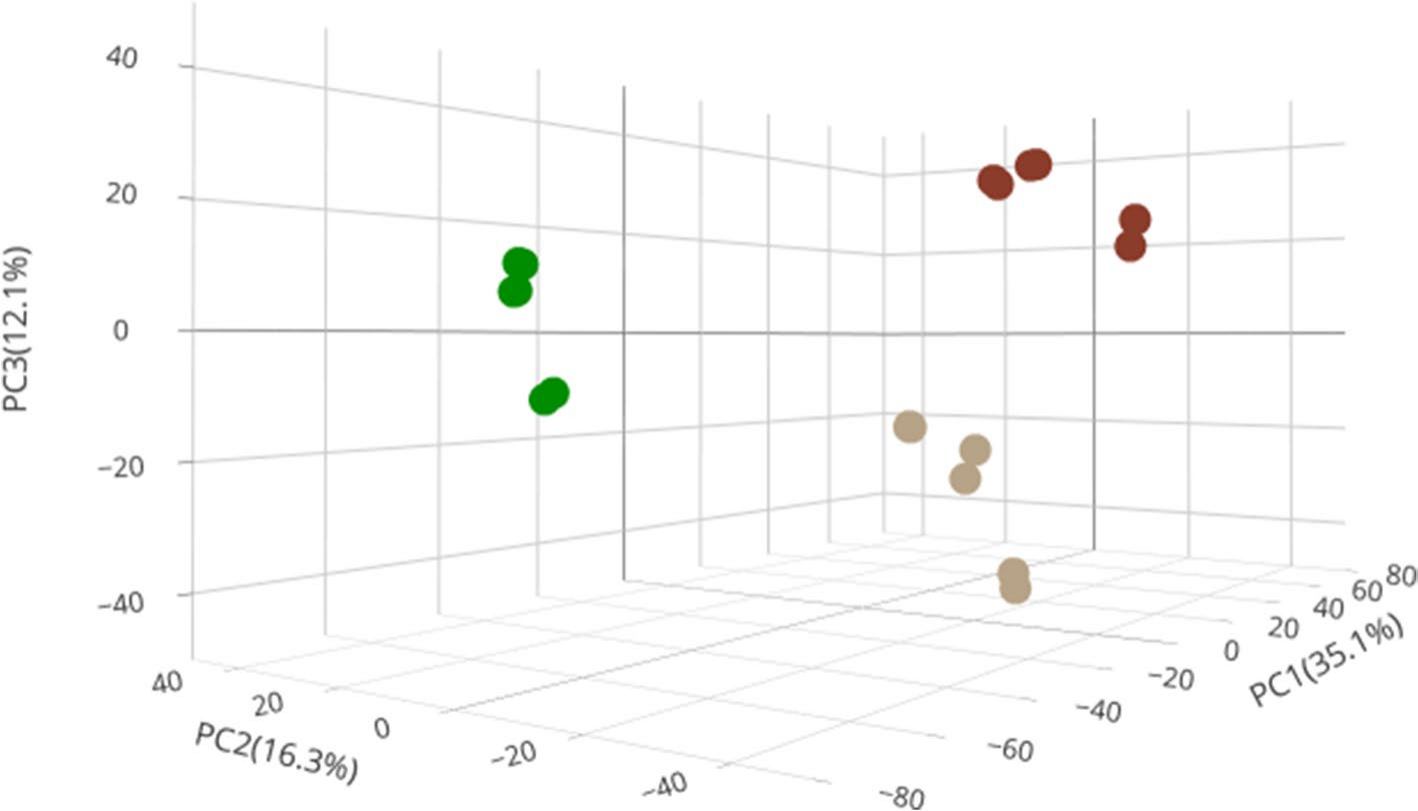
Principal Component Analysis (PCA) of the proteome data in a 3D-plot. Group of sample sets that are close together are highly correlated in terms of the proteome profile for each condition while the group of distant sample sets is less correlated. Different treatment conditions are indicated by different-colored circles; Gefitinib-treated samples are marked with a brown dot, R9VH36-treated samples with a green dots, and the control-samples with red dots

LC–MS/MS based proteomics is a very sensitive method for protein identification and quantification. Proteins with their relative abundance having a *q*-value less than 0.05 between 18 LC-runs are considered statistically significant differentially expressed. A total of 6,626 differentially expressed proteins were successfully identified by LC–MS/MS (supplementary 2) among three sets of experimental groups. Proteomics analysis allowed us to access the extent of the changes in protein abundances and the degree of variance in the proteome profiles in different treatment conditions. Principal component analysis (PCA) was used to reduce a large data dimension to a smaller number of groups for easier visualization and interpretation. We performed PCA plotting by comparing any of the 3 sample groups in the 18 LC-runs and evaluated intra- and inter-group variations in gefitinib, R9VH36, and control. The PCA resulted were consistent that each group was distinguished. The plotting of PC1 to PC3 determined that the control group (red dots) was closely related to the gefitinib group (brown dots) than R9VH36 (green dots) (Fig. 2).

The R9VH36 group clustered in the left region while gefitinib and control groups clustered in the right region. Although the intra-group had slightly larger variations, which may be due to the small sample size, or largely uncontrollable factors, such as treatment variation, and other pre- and post-sample collection variance. However, PCA analysis of all identified proteins displayed the complete separation of control and gefitinib and R9VH36 groups. Overall, PCA indicated that the protein profile was predominantly changed.

**Fig. 3:**
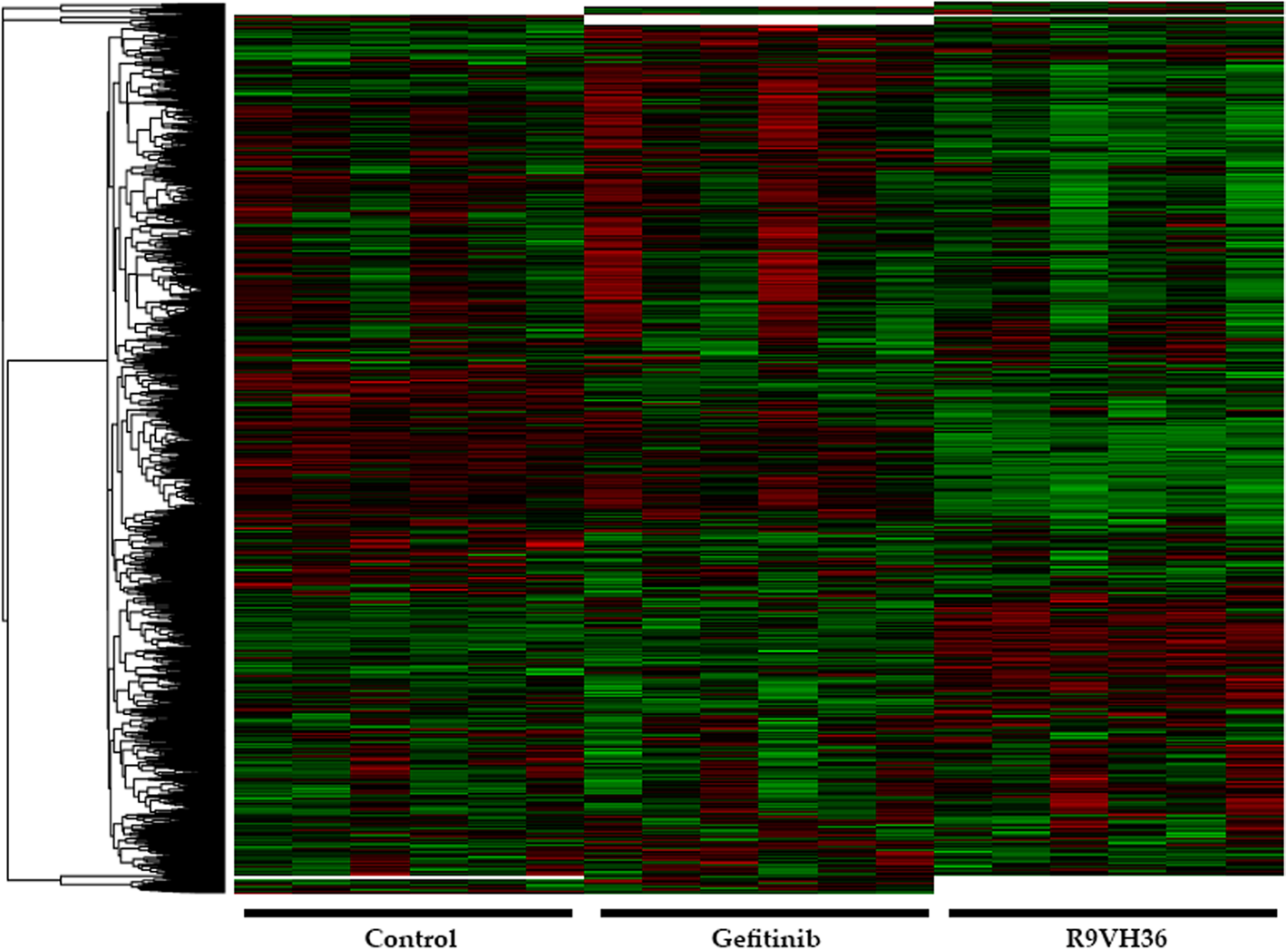
The heatmap with hierarchical clustering of differentially expressed proteins. Color key expression: Green and red represent the lower-and-higher differential abundance, respectively

The difference of protein expressions among these experimental groups was clustered to hierarchical clustering as represented in the heatmap in Fig. 3. The columns show the relative protein expression. The rows present significantly expressed proteins with a maximum distance = 1.0. The heatmap depicts their relative abundance level by the color coding (lower- and higher-expression level: green and red, respectively).

**Fig. 4:**
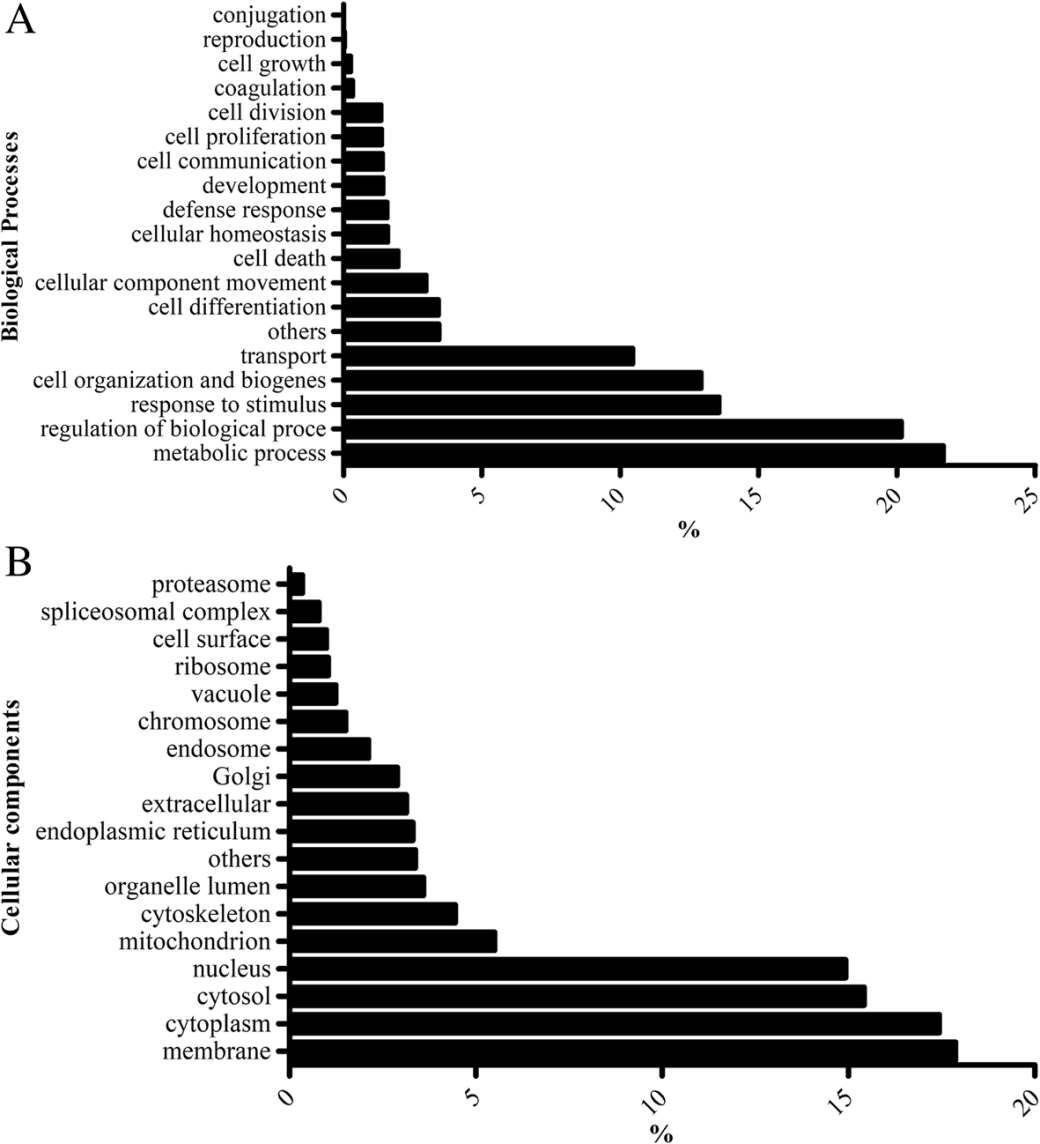
Comparison of the relative abundance of proteins according to gene ontology terms. Gene ontology categories of biological process (**A**) and cellular component (**B**) GO-domain were used for annotation of the identified proteins. The bars represent the relative abundance of GO categories

To classification the overall protein task in the cellular machinery, the proteome dataset was subjected to Gene ontology analysis according to biological function as illustrated in Fig. 4 via bar chart. Based on biological process, the identified proteins were varied and divided into 19 functional groups, mainly comprised metabolic process (21.68%) and regulation of biological process (20.16%) (Fig. 4A). The identified proteins were distributed by location relative to the cellular components and structure irregularly, the majority on the membrane (17.88%) (Fig. 4B).

To study the difference of protein markers between the gefitinib and R9VH36 treatment groups, a strict criterion was performed to obtain confidential data. Only proteins quantified with at least 3 unique peptides and differential expression ratio > 20 were considered, there were 15 proteins as shown in Table 1.

**Table 1:** Proteomics data with differential expression ratio > 20 between gefitinib and R9VH36 groups

Accession	Protein name	*q*-value	Ratio: Gef/Con	*p*-value: Gef/Con	Ratio: R9VH36/Con	*p*-value: R9VH36/Con
Q8IVF2	Protein AHNAK2	0	100	1.78E^−16^	100	1.51E^−16^
Q9BVA0	Katanin p80 WD40 repeat-containing subunit B1	0.001	100	1.78E^−16^	100	1.51E^−16^
P02042	Hemoglobin subunit delta	0	0.728	3.01E^−02^	25.327	1.51E^−16^
Q5VZ66	Janus kinase and microtubule-interacting protein 3	0.029	1.076	9.05E^−01^	21.564	1.51E^−16^
O15397	Importin-8	0	0.103	1.78E^−16^	21.246	1.51E^−16^
Q13315	Serine-protein kinase ATM	0	1.144	9.40E^−01^	0.01	1.51E^−16^
P54802	Alpha-N-acetylglucosaminidase	0	1.444	5.28E^−01^	0.01	1.51E^−16^
Q9HD67	Unconventional myosin-X	0	1.321	2.79E^−01^	0.01	1.51E^−16^
Q99707	Methionine synthase	0.002	1.307	4.10E^−01^	0.01	1.51E^−16^
Q9NX05	Constitutive coactivator of PPAR-gamma-like protein 2	0	1.305	7.79E^−01^	0.01	1.51E^−16^
Q969X5	Endoplasmic reticulum-Golgi intermediate compartment protein 1	0	1.26	3.73E^−01^	0.01	1.51E^−16^
A6NED2	RCC1 domain-containing protein 1	0	1.199	7.47E^−01^	0.01	1.51E^−16^
O15379	Histone deacetylase 3	0	0.935	9.76E^−01^	0.01	1.51E^−16^
O43156	TELO2-interacting protein 1 homolog	0	0.837	8.66E^−01^	0.01	1.51E^−16^
Q5NDL2	EGF domain-specific O-linked N-acetylglucosamine transferase	0	0.01	1.78E-^16^	0.01	1.51E^−16^

According to Table 1, in comparison between R9VH36 and control, 5 proteins were over-expressed and 10 proteins were under-expressed. The detailed description and function of these proteins are presented in supplementary data 2. The biological functions of these proteins vary from being a transportation protein, metabolic process, angiogenesis, and the development of keratinocytes. Noticeably, gefitinib and R9VH36 treatment on SW480 cells in various biological functions and cellular components.

## Discussions

The R9VH36 have previously reported that they were able to decrease the viability of the EGFR-driven cancer cell, A549, in a nanomolar range which is. Surprisingly, it also did not affect MCF-7 and HepG2 which are EGFR-negative cancer cells. Moreover, Vero cell was used as normal cell control for biocompatible examining. The result also demonstrated VHs/VHH format did not affect normal cells as well [20]. Herein, SW480 cells which is similar character cell line to A549 cells. SW480 is an EGFR-driven cancer cell and harboring KRAS mutant (G12V) instead of G12S in A549. To monitor proteomic profiling of SW480 after R9VH36 treatment compared to TKI, gefitinib has been used as a targeting EGFR inhibitor which acts as ATP-analogue to prevent ATP at the catalytic site of EGFR-TK leading to reduce EGFR phosphorylation. On the other hand, epitope mapping revealed that R9VH36 was able to bind closely to catalytic site of EGFR-TK. Therefore, a comparison of R9VH36 and gefitinib might reveal the different mechanisms of both inhibitors.

To explore the effect of R9VH36 and gefitinib on SW480 cells, differential proteome analysis was conducted. Generally, proteomics analysis using LC–MS/MS encompasses the following major steps including protein digestion, peptide fractionation using LC, and subsequent data acquisition using MS. A proteomics data set is used to identify and quantify proteins that are different among sample conditions. Intermediate results including total ion count (TIC) and peptide–spectrum matches (PSM) are commonly available as well. Subsequently, it is highly recommended to carry out a QC assessment to avoid bias results for downstream analysis. The QC of this experiment was done and clarified. This approach used qualitative and quantitative multiplex analysis in different batches to monitor individual variation caused by production techniques, starting material, etc. [23, 24]. Quantification of protein abundances included comparisons of peak intensities across multiple LC–MS runs. Integration of the LC–MS runs including LC fractionation condition and LC–MS/MS data revealed an alignment of the TIC of all LC-runs were consistent and reproducible (supplementary data 1). TIC for different samples was acquired from LC–MS/MS exhibited peptide ions intensities fluctuation. In the first LC-runs of control, gefitinib and R9VH36 groups revealed the highest TIC peak is at about 9 min for all replications.

Maximum peptide mass deviation is an operationally important parameter related to mass accuracy in a proteomics dataset [25]. Due to the high-solution LC–MS, maximization specificity of the peptide mass deviation was strictly set at 20 ppm. The narrow peptide mass deviation cut-off value acts as a filter that directly reduces the number of potential false-positive peptides. The analysis revealed 86.29%% of identified peptides had a mass deviation of ≤ 10 ppm suggested that the major peptides achieved a good mass precision. The majority of tryptic peptides (80.42%) were between 10 and 25 amino acid residues long. In addition, 72.93% of tryptic peptides also revealed no missed cleavage peptides. We observed roughly 23% of the tryptic peptides containing one or more tryptic sites, which agreed with the known properties of the tryptic digestion approach [26]. The signal fluctuation from short peptide length (< 6 amino acid residues) and a high level of missed cleavage sites can affect the quality of protein identification and quantification.

Measurement of the proportion of peptide FDR in proteomics analysis is important to assess and maintain the solid quality of protein identifications. The general approach to measure the FDR in the analysis is target-decoy search [27]. This approach assigns a score to each PSM based on the frequency of false discovery for peptide identifications. This algorithm uses a *q*-value for measurement FDR at the protein level in this analysis. A total of 83.54% of identified proteins has FDR ≤1% which is defined as confidently identified proteins.

**Fig. 5:**
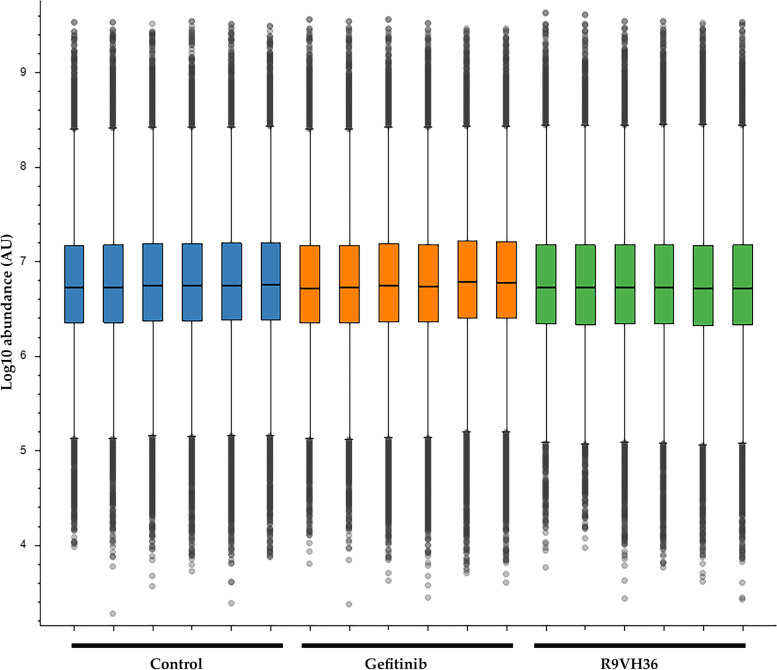
Peptide level normalization. Box-and-whisker plots showed intensities distribution of the identified peptides per LC–MS run. The plots denote the lowest datum still within 1.5 × IQR of the Q3 values, and the highest datum still within 1.5 × IQR of the Q3 values. The median values indicated as a horizontal line inside each box. The points lying beyond the boxes were considered as outliers. Y-axis is the log10 peptide abundance and X-axis is LC–MS run. Different treatment conditions are indicated by different-colored bars; Gefitinib-treated samples are marked with an orange bar, R9VH36-treated samples with a green bar, and the control-samples with blue bar

These results suggested that the sample preparation and proteolysis were acceptable. It was implied that the results were likely to be optimal surrogates for proteome quantitation, particularly for the approaches seeking biochemical pathways. The normalization of protein expression data requires a dataset correction. To figure out this issue, peptide-level normalization was conducted to reduce the non-biological related variations and make the results reliable for downstream analysis. After normalization, the situation is changed, and an interquartile range (IQR) of total peptide abundances of all samples are nearly equal as illustrated in Fig. 5.

The normalization decreased intragroup and inter groups variation measured as log10 (peptides abundance). Because of the raw data containing missing values, especially for those with relatively low abundance in the cellular system. They are common in most LC–MS/MS analyses due to the randomness in sampling during the analysis [28]. The limitation from these missing values can be fixed by removing or including based on normalization algorithm and statistical analysis of all quantitated proteins.

The Protein AHNAK2 and Katanin p80 WD40 repeat-containing subunit B1 were expressed at the highest level in the R9VH36 group and gefitinib. AHNAK2 is a protein for neuroblast differentiation and cell migration and Katanin p80 WD40 repeat-containing subunit B1 serves as the reorganization of cellular microtubule in the cells, which they did not have any specific molecular mechanism in SW480 cells. AHNAK2 gene was upregulated in pancreatic cancer but the molecular function of this protein is inconclusive [29]. Katanin p80 WD40 repeat-containing subunit B1 facilitates a complex which severs microtubules in an ATP-dependent manner, promotes rapid reorganization of cellular microtubule arrays. Together with other KTN80s, regulates cell elongation by modulating MT organization. These results suggested us two proteins are major actors similarly affected by R9VH36 and gefitinib.

**Fig. 6:**
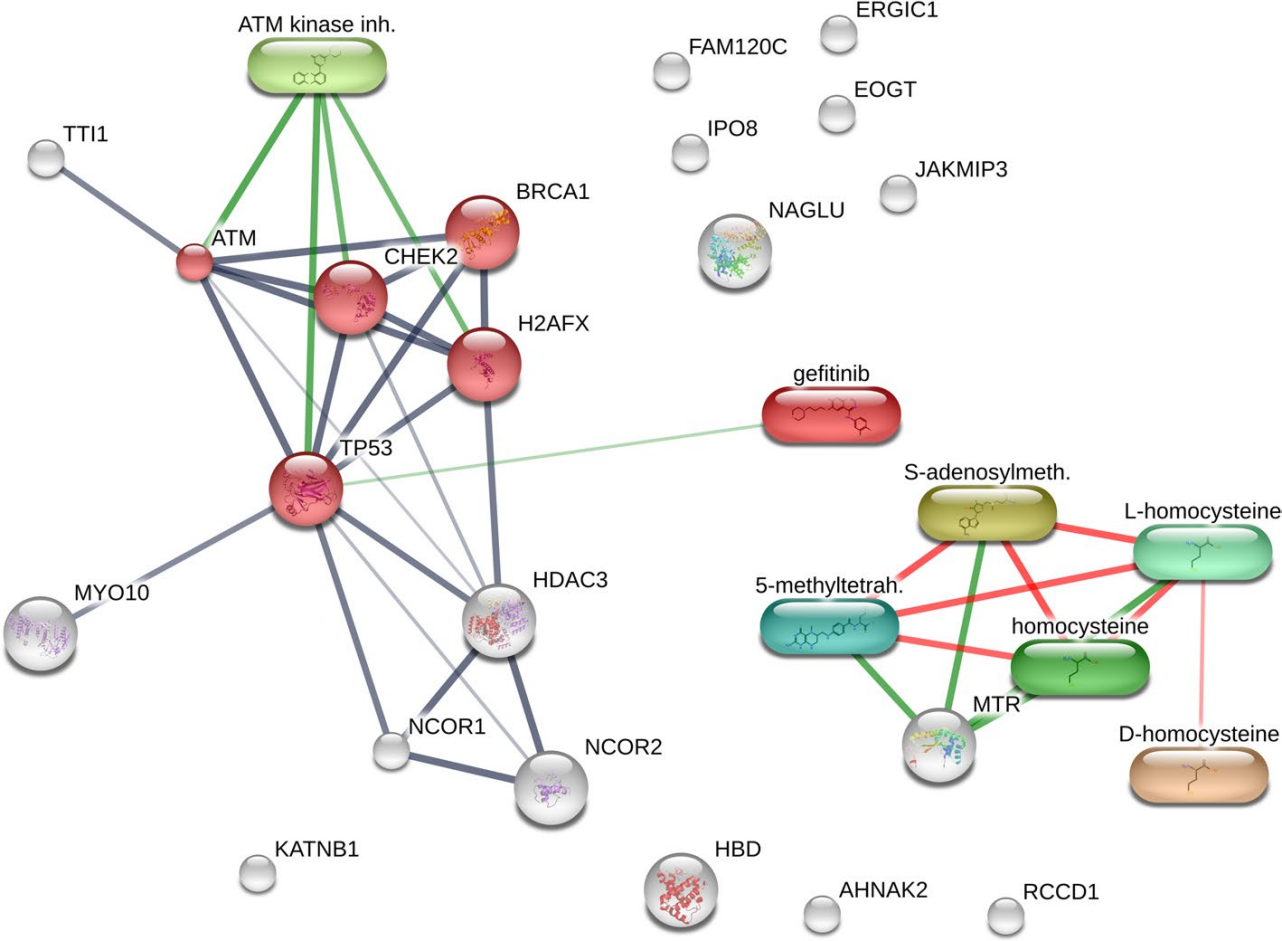
Interaction network of gefitinib and the differential expressed proteins. The interactions were represented in different colors including protein–protein interactions are shown in grey, gefitinib-protein interactions in green, and interactions between other chemicals in red

Among 15 proteins, there are 8 proteins in R9VH36 that exhibited opposite expression directions when compared to gefitinib. Generally, proteins do not act independently. They interact as either transient or stable complexes with their partner proteins or other macromolecules. We used the STRING protein–ligand prediction tool to clarify the biochemical mechanism of R9VH36. A total of 15 proteins with gefitinib were imported and searched with *Homo Sapiens database*. The ligand–protein interaction networks were shown in Fig. 6.

Functional enrichment analysis revealed that the highest biological process affected by VH36 and gefitinib was the DNA-damage checkpoint. To complete the network, 6 proteins (NCOR1, TP53, CHEK2, H2AFX, NCOR2, BRCA1) and 4 ligands (homocysteine, 5-methyltetrahydrofolate, S-adenosylmethionine, ATM-kinase inhibitor) were recruited in this analysis. Among 5 proteins (red node) as illustrated in Fig. 6 that classified in DNA-damage checkpoint (FDR = 0.0026), Serine-protein kinase ATM (Q13315) was shown the direct interaction with CHEK2, TP53, BRCA2, and H2AFX. The expression of this protein was down-regulated in the VH36 condition, but it was up-regulated in the gefitinib condition. Serine-protein kinase ATM can activate signaling cascade including double-strand breaks (DSBs), apoptosis, and genotoxic stresses. Another protein was the constitutive coactivator of PPAR-gamma-like protein 2 (Q9NX05), it was down-regulated in R9VH36 condition, but it was up-regulated in gefitinib condition. The PPAR-gamma-like protein 2 acts mainly as regulation of transmembrane proteinases, EGFR transactivation, stress response, interaction with Wnt/β-catenin, and cell proliferation. Down-regulation of these proteins could affect the cellular stresses and lead to cell death, these results might explain why the R9VH36 had lower cellular toxicity than gefitinib.

## Conclusions

Nanobody (R9VH36) significantly reduced SW480 cell viability by more than 2 folds compared to gefitinib, a well-known EGFR inhibitor drug. The proteomics explored those 6,626 proteins had different expressions between VH36 and gefitinib. Comparison between R9VH36 and control dominant proteins, there were 8 proteins (P02042, Q13315, P54802, Q9HD67, Q99707, Q9NX05, Q969X5, and A6NED2) in R9VH36 exhibited opposite expression direction when comparing to gefitinib. These proteins are involved in DNA-damage checkpoint processes. This information could lead us to a broader understanding of the effect of R9VH36 in SW480 cells, thus supporting its value for being a novel therapeutic strategy in colon cancers.

## Supplementary Information

**Additional file 1.**

**Additional file 2.**

**Additional file 3: Supplementary Figure 1.** Growth inhibitory effects of drug and nanobody treatments in the NIH/3T3 cell lines (EGFR-negative mouse fibroblast). **Supplement Figure 2.** Growth inhibitory effects of nanobody treatments on the EGFR-positive KRAS mutations colorectal cancer, SW620(G12V) and HCT116(G13D).

## Data Availability

All data generated or analyzed during this study are included in this published article and its supplementary information files.
