# Supplementary material for: A novel nanobody as therapeutics target for EGFR-positive colorectal cancer therapy: exploring the effects of the nanobody on SW480 cells using proteomics approach
Source: Proteome Sci. 2022 May 16;20:9. doi: 10.1186/s12953-022-00190-6 (PMC9109347; doi:10.1186/s12953-022-00190-6)
Supplement: Supplementary file 1 — Additional file 1. [file 12953_2022_190_MOESM1_ESM.docx]

**Supplementary material**

**Supplementary data 1**

TIC Quality control assessment. LC-MS profiles analysis of a cell line lysate in single runs of 145 minutes. (A) Total ion count of SW480 cells lysate. Unit of X- axis and Y-axis are minutes and % intensity abundance, respectively.

| **Condition** | **Bio-replication** | **Technical replication** | **TIC** |
| --- | --- | --- | --- |
| Control | 1 | 1 |  |
|  |  | 2 |  |
|  | 2 | 1 |  |
|  |  | 2 |  |
|  | 3 | 1 |  |
|  |  | 2 |  |
| Gefitinib | 1 | 1 |  |
|  |  | 2 |  |
|  | 2 | 1 |  |
|  |  | 2 |  |
|  | 3 | 1 |  |
|  |  | 2 |  |
| R9VH36 | 1 | 1 |  |
|  |  | 2 |  |
|  | 2 | 1 |  |
|  |  | 2 |  |
|  | 3 | 1 |  |
|  |  | 2 |  |
