# Supplementary material for: A novel nanobody as therapeutics target for EGFR-positive colorectal cancer therapy: exploring the effects of the nanobody on SW480 cells using proteomics approach
Source: Proteome Sci. 2022 May 16;20:9. doi: 10.1186/s12953-022-00190-6 (PMC9109347; doi:10.1186/s12953-022-00190-6)
Supplement: Supplementary file 3 — Additional file 3: Supplementary Figure 1. Growth inhibitory effects of drug and nanobody treatments in the NIH/3T3 cell lines (EGFR-negative mouse fibroblast). Supplement Figure 2. Growth inhibitory effects of nanobody treatments on the EGFR-positive KRAS mutations colorectal cancer, SW620(G12V) and HCT116(G13D). [file 12953_2022_190_MOESM3_ESM.docx]

**Supplementary Figure**

**
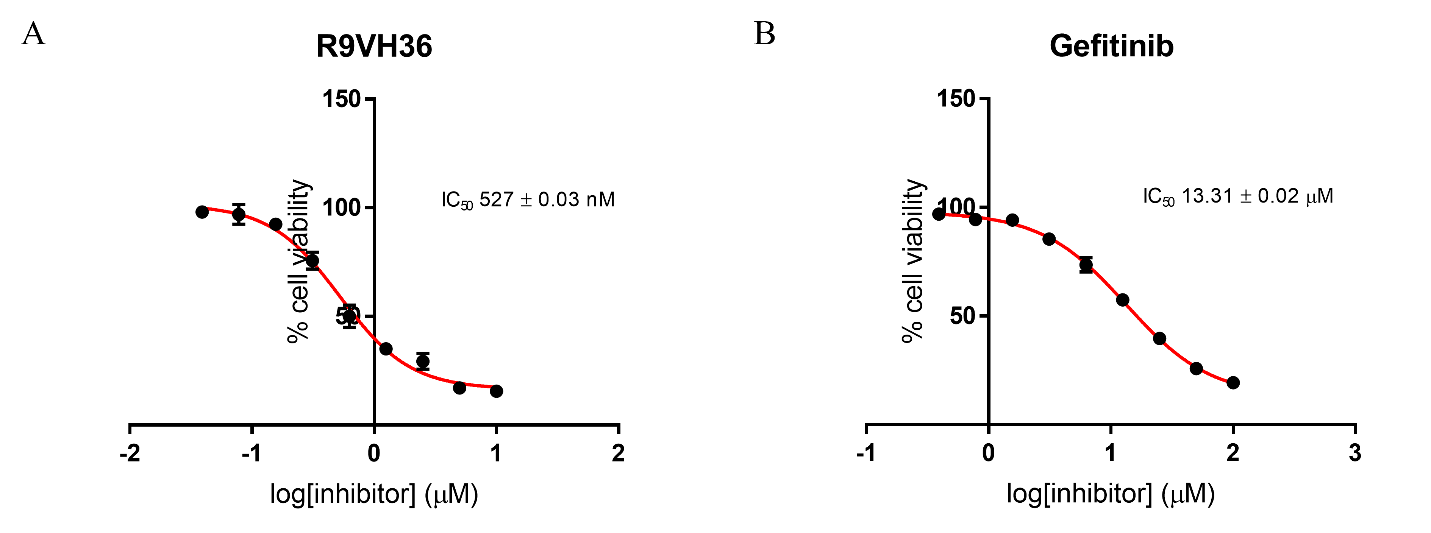
**

Supplementary Figure 1. Growth inhibitory effects of drug and nanobody treatments in the NIH/3T3 cell lines (EGFR-negative mouse fibroblast). The cells were treated with various concentrations of R9VH36 (A) and gefitinib (B) for 3 days. The results are expressed as mean ± SD of triplicate experiments.


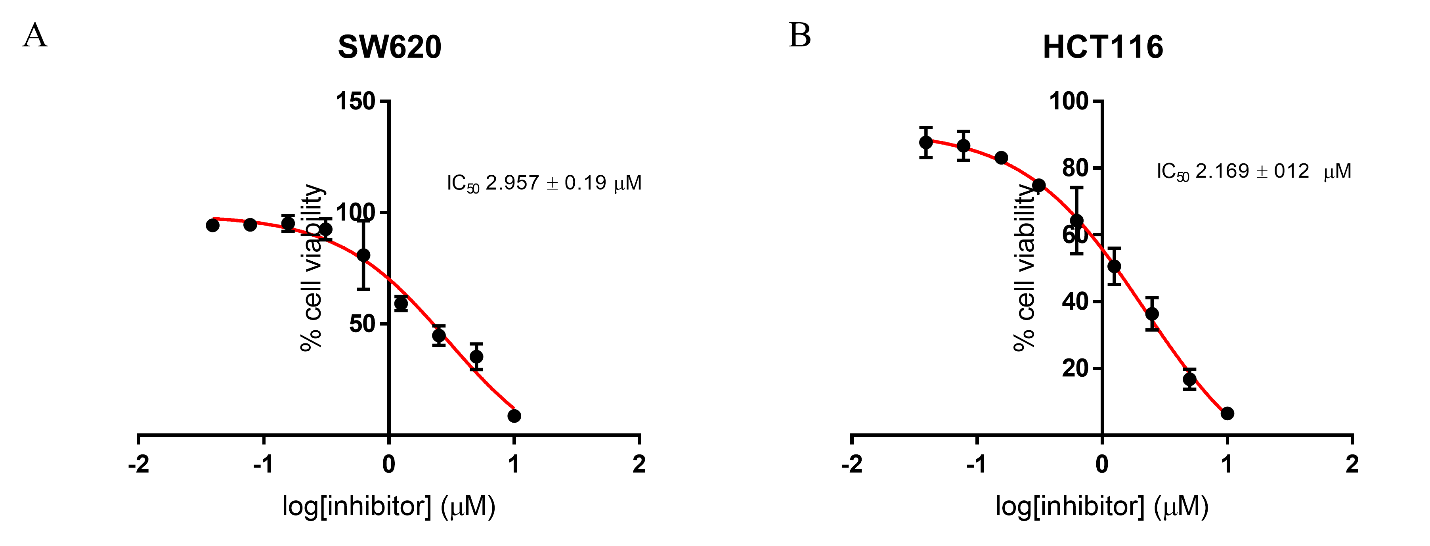


Supplement Figure 2. Growth inhibitory effects of nanobody treatments on the EGFR-positive KRAS mutations colorectal cancer, SW620(G12V) and HCT116(G13D). The various concentrations of R9VH36 treatment on SW620(A) and HCT116 (B) for 3 days. MTT reagent was added for the last 3 h, the IC_50_ estimated using GraphPad Prism (GraphPad Software Inc., San Diego, CA, USA). The results are expressed as mean ± SD of triplicate experiments.
